# Different roles of endothelial cell-derived fibronectin and plasma fibronectin in endothelial dysfunction

**DOI:** 10.55730/1300-0144.5735

**Published:** 2023-10-25

**Authors:** Xiaoxin LUO, Weixiong JIAN

**Affiliations:** 1 Department of Traditional Chinese Medicine Diagnostics, Faculty of Traditional Chinese Medicine, Hunan University of Chinese Medicine, Changsha, China; 2 Department of National Key Discipline of Traditional Chinese Medicine Diagnostics and Hunan Provincial Key Laboratory, Faculty of Traditional Chinese Medicine, Hunan University of Chinese Medicine, Changsha, China

**Keywords:** Endothelial cell activation, endothelial dysfunction, inflammation, atherosclerosis, fibronectin

## Abstract

**Background/aim:**

Atherosclerosis is significantly influenced by endothelial cell activation and dysfunction. Studies have demonstrated the substantial presence of fibronectin (Fn) within atherosclerotic plaques, promoting endothelial inflammation and activation. However, cellular Fn (cFn) secreted by various cell types, including endothelial cells and smooth muscle cells, and plasma Fn (pFn) produced by hepatocytes. They are distinct forms of Fn that differ in both structure and function. The specific contribution of different types of Fn in promoting endothelial cell activation and dysfunction remain uncertain. Therefore, this study aimed to investigate the respective roles of pFn and endothelial cell-derived Fn (Fn^EC^) in promoting endothelial cell activation and dysfunction.

**Materials and methods:**

Initially, endothelial cell injury was induced by exposing the cells to oxidized low-density lipoprotein (ox-LDL) and subsequently we generated a mutant strain of aortic endothelial cells with Fn knockdown (Fn^EC-KD^). The impact of the Fn^EC-KD^ arel the addition of pFn on the expression levels of inflammatory factors, vasoconstrictors, and diastolic factors were compared.

**Results:**

The results showed that the Fn^EC-KD^ significantly inhibited ox-LDL-induced intercellular adhesion molecule 1 (ICAM-1, p < 0.05), vascular cell adhesion molecule (VCAM-1, p < 0.05), and endothelin (p < 0.05) expression, and nuclear factor kappa-B (NFκB, p < 0.05) activation. These results implied that Fn^EC-KD^ inhibited both endothelial cell activation and dysfunction. Surprisingly, the addition of pFn significantly inhibited the ox-LDL-induced ICAM-1 (p < 0.05), VCAM-1 (p < 0.05), and endothelin (p < 0.05) expression and NFκB (p < 0.05) activation. Implying that pFn inhibits endothelial cell activation and dysfunction. Additionally, the study revealed that ox-LDL stimulation enhanced the production of excessive nitric oxide, leading to severe endothelial cell damage.

**Conclusion:**

Aortic Fn^EC^ promotes endothelial cell activation and endothelial dysfunction, whereas pFn inhibits ox-LDL-induced endothelial cell activation and endothelial dysfunction.

## 1. Introduction

Atherosclerosis (AS) is a chronic inflammatory artery disease that has emerged as a major public health concern around the world [1,2]. Endothelial cell dysfunction is defined by increased inflammatory gene expression or decreased diastolic and contractile function [3], whereas endothelial cell activation is characterized by increased inflammatory gene expression and permeability, both of which are thought to be major causes of AS [4]. With the formation of AS, circulating low-density lipoproteins (LDLs) undergo oxidative modifications and become oxidized LDLs (ox-LDLs) as they traverse the compromised endothelial cell barrier [5], and ox-LDL then promotes endothelial cell activation [6]. Through α5β1 [7] or toll-like receptor 4 (TLR4) [8–10], ox-LDL activates inflammatory transcription factors such as nuclear factor kappa-B (NFκB) in endothelial cells and causes cells to secrete inflammatory factors such as interleukin 1 (IL-1), intercellular adhesion molecule 1 (ICAM-1), vascular cell adhesion molecule (VCAM-1) [11], and p-selectin [4]. This process facilitates the activation of endothelial cells.

Moreover, the deposition of fibronectin (Fn), known to contribute to endothelial cell activation [6], was observed in the region predominantly occupied by atherosclerotic plaques [12]. However, collagen IV and laminin constitute nearly all of the extracellular matrix of healthy artery endothelial cells, whereas Fn is scarce [13]. This implies that Fn may be involved in the pathological development of AS. Following that, the researchers conducted extensive research. The formation of a malondialdehyde-Fn (MDA-Fn) complex, induced by aldehydes generated through oxidation in the body, has been proposed. The immune response triggered by MDA-Fn is believed to contribute to the clearance of dysfunctional proteins from plaques, thereby reducing vascular inflammation [14]. However, additional research has indicated that Fn promotes vascular inflammation. Fn has been shown to disrupt the antiinflammatory cyclic adenosine monophosphate/protein kinase A pathway, resulting in the dysregulation of NFκB inhibition [15]. Consequently, the expression of inflammatory genes, including ICAM-1, VCAM-1, E-selectin, and IL-6, is increased [16–20]. Cellular Fn (cFn) and plasma Fn (pFn) are the 2 types of Fn. cFn is a common extracellular matrix component synthesized by smooth muscle cells, endothelial cells, and other cells, whereas pFn is abundant in the bloodstream and secreted by hepatocytes [21]. The differential roles of Fn from various sources in endothelial cell activation and endothelial dysfunction are currently under investigation. The objective of this study was to assess the impact of pFn and aortic endothelial cell-derived Fn (Fn^EC^) on endothelial cell activation and dysfunction.

Given that endothelial cell activation and dysfunction are fundamental drivers of AS, this study established an endothelial cell injury model utilizing rat aortic endothelial cells and ox-LDL. In endothelial cells with Fn knockdown (Fn^EC-KD^) or cells exposed to pFn, the expression of inflammatory, vasoconstrictor, and diastolic factors was then investigated. The findings effectively elucidated the respective roles of pFn and Fn^EC^ in endothelial function.

## 2. Materials and methods

### 2.1. Cell culture

Rat aortic endothelial cells (AECs; Procell Life Science & Technology Co., Ltd., Wuhan, China) were cultured in rat AEC complete medium (Procell; Lot No. CM-R075), which contained M199 medium, 5% phosphate buffered saline, 1% penicillin/streptomycin solution, heparin, hydrocortisone, endothelial growth factors, and other growth factors (epidermal growth factor, basic fibroblast growth factor, insulin-like growth factor, and vascular endothelial growth factor). The experiments used AECs from the third to fifth generation. All of the experiments were carried out in 6-well cell culture plates with the rat AECs complete medium.

### 2.2. Knockdown of Fn

**Figure 1:**
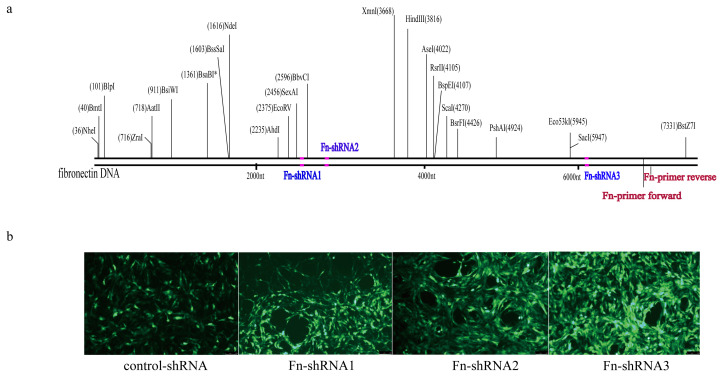
(a) Image of the positions of 3 shRNAs and primer targeting Fn. Numbers indicate nucleobase sorting. Pink markers are the locations of the shRNAs. (b) Images of RAECs transfected with Fn-targeted shRNA or control shRNA (MOI = 500). Green fluorescence is caused by GFP, which is carried by adenovirus. Representative images are shown at 200X magnification.

The recombinant adenovirus of short hairpin RNA (shRNA) targeting rat Fn1 (NM 019143.2) was used (Hanheng Biotechnology Co., Ltd., Shanghai, China). The AECs were transfected separately with 500 multiplicity of infection (MOI) adenovirus containing Fn shRNA1, Fn shRNA2, Fn shRNA3, or control shRNA (the sequences and position of the shRNA constructs in the transcript are shown in Table 1 and Figure 1a), corresponding to the Fn-KD1, Fn-KD2, Fn-KD3, and control groups, respectively.

The transfection method provided in the manufacturer’s instructions for the adenovirus was followed: the cells were cultured with shRNA adenoviral constructs that were infected with green fluorescent protein (GFP) mixed with 1 mL of culture medium for 4 h. The other 1 mL of the culture medium was then added, and the transfection was continued for 2 h. The entire transfection process took about 6 h. The medium with adenovirus was then replaced with fresh medium. The AECs were observed (see Figure 1b) and Fn mRNA was detected by fluorescence quantitative polymerase chain reaction (qPCR) 48 h after transfection in a constant temperature cell culture incubator at 37 °C and 5% CO_2_.

Fn shRNA1 was chosen and transfected into 80% confluent AECs to generate Fn^EC-KD^. Following a 48-h recovery period after transfection, ox-LDL was added to the AECs for 24 h to complete the endothelial cell injury model and index tests. This group was designated as the Fn^EC-KD^ group. Moreover, the negative control (NC) group was transfected with control-shRNA adenovirus, while the ox-LDL group was subjected to a 24-h treatment with ox-LDL when the cell confluency reached 80%. A blank group served as the control with no intervention, representing normal cultured cells.

### 2.3. Determination of pFn concentration

A total of 5 mg of lyophilized human pFn powder (pFn, Lot NO. 10838039001, Sigma-Aldrich Chemical Co., St. Louis, MO, USA) was dissolved in ultrapure water to obtain a concentration of 1 mg/mL at 37 °C. The experiment began with 3 concentrations of pFn solution at 5, 10, and 20 μg/cm^2^ and 1 mL of ultrapure water in advance to coat the 6-well cell culture plates for 24 h, corresponding to the Fn5, Fn10, Fn20, and ox-LDL groups. When the cell confluence reached 80%, ox-LDL intervention was added for 24 h. A blank group was also created with no intervention for normal cultured cells.

### 2.4. Determination of ox-LDL concentration

The experiment was initiated utilizing a 96-well cell culture plate to evaluate various concentrations of ox-LDL (YB-002; Yiyuan Biotechnologies, Guangzhou, China), aiming to identify the optimal concentration for establishing an endothelial cell injury model. With 100 μL of cell suspension per well, the cell culture density was 5 × 10^4^/mL. When cell confluency reached 80%, the ox-LDL intervention was added at final concentrations of 0, 40, 60, 80, 120, and 160 μg/mL, and 4–5 replicate wells for each group. The negative control group received a similar volume of PBS. The ox-LDL stock solution was diluted in CM-R075 medium, and cell viability was assessed 24 h after the ox-LDL intervention using the cell count kit 8 (CCK8).

### 2.5. Cell viability

Subsequently, the CCK-8 reagent (BioSharp, Hefei City, Anhui Province, China) was added for a 2-h incubation period, followed by the measurement of the absorbance optical density (OD) at 450 nm using an enzymatic marker. The following formula was used to calculate the cell viability: cell viability (%) = [OD (spiked) – OD (blank)] / [OD (0 spiked) – OD (blank)]. The endothelial cell injury model was established by selecting the optimal concentration of ox-LDL based on cell viability and growth assessment.

### 2.6. Fluorescence quantitative PCR

mRNA was extracted from the AECs using the SteadyPure Universal RNA Extraction Kit (AG21017, Accurate Biotechnology (Human) Co. Ltd. Changsha City, Human Province, China. To make first-strand DNA, the Evo M-MLV RT Kit (AG11705, Accurate Biotechnology (Human) Co. Ltd.) was used. On a Roche LightCycler, quantitative fluorescent PCR was performed using the SYBR Green Premix Pro Taq HS qPCR kit (AG11701, Accurate Biotechnology (Human) Co. Ltd.). The following process was followed for amplification. The first stage of the experiment involved a 30-s incubation at 95 °C. This was followed by the second stage, which consisted of 40 cycles of temperature changes (5 s at 95 °C, 30 s at 60 °C). The third stage involved dissociation. For detailed information regarding the primer sequences and their corresponding positions in the Fn transcripts, see Table 2 or Figure 1a.

### 2.7. Immunoblotting and immunocytochemistry

Cells were lysed on ice with a lysis mix containing 98% radioimmunoprecipitation assay lysis buffer, 1% phenylmethylsulfonyl fluoride, and 1% phosphatase inhibitor. The lysates were transferred to a polyvinylidene difluoride membrane (Merck Millipore, Burlington, MA, USA). Before incubation with primary antibodies, the membranes were blocked for 1 h with 5% non-fat dry milk. ImageJ software (US National Institutes of Health, Bethesda, MD, USA) was used to perform the densitometry. The antibodies included beta actin antibody (1:4000, Proteintech Group, Inc., Rosemont, IL, USA) and NFκB p65 (1:2000, Proteintech Group, Inc.).

The cells were fixed in 4% paraformaldehyde for 30 min before being permeabilized in 0.3% Triton X100 (T8200, Beijing Solebro Technology Co., Beijing, China) for 10 min. Cells were rinsed and then blocked for at least 1 h with 10% goat serum (Beijing Solebro Technology Co., SL038). The cells were then stained with primary antibodies (1:100) overnight at 4 °C before being exposed to fluorescently labeled secondary antibodies. Then, 4′,6-diamidino-2-phenylindole was used to stain the nuclei. Finally, the stain was examined using a Leica microsystem with a Leica dfc900GT camera and imager configuration (Leica Microsystems, Wetzlar, Germany). Cell counts were performed for each field of view and calculated according to the following formula: cell positivity rate = positive cells / total cells × 100%. NFκB p65 (1:2000, Proteintech Group, Inc.), CoraLite594-conjugated goat antirabbit IgG (H+L) (SA00013-4, Proteintech Group, Inc.) were among the antibodies used.

### 2.8. ELISA and biochemical assay

Commercially available ELISA kits were used to examine the cell culture supernatants for VCAM-1 (RX302198R, Quanzhou Ruixin Biological Techology Co. Ltd. Quanzhou City), ICAM-1 (RX302262R, Quanzhou Ruixin Biological Techology Co. Ltd.), prostacyclin I2 (PGI2, RX302410R), p-selectin (RX302948R, Quanzhou Ruixin Biological Techology Co. Ltd.), endothelin (ET, RX302459R, Quanzhou Ruixin Biological Techology Co. Ltd.). The nitric oxide (NO) assay was carried out according to the kit protocol using a commercially available NO (with the nitrate reductase method) assay kit (RXWB0246-96, Quanzhou Ruixin Biological Techology Co. Ltd.).

### 2.9. Statistical analysis

The IBM SPSS Statistics for Windows 25.0 (IBM Corp., Armonk, NY, USA) was used to perform statistical comparisons between the groups. The statistical significance of the parameters was assessed using student’s t test (2-tailed) for comparisons of 2 groups or 1-way analysis of variation (ANOVA), followed by a post hoc test with least significant difference (LSD) or Dunnett’s T3. The data was presented as the mean standard deviation. Statistical significance was accepted as p < 0.05.

## 3. Results

### 3.1. Fn derived from the AECs promotes ox-LDL-induced endothelial cell inflammation

**Figure 2:**
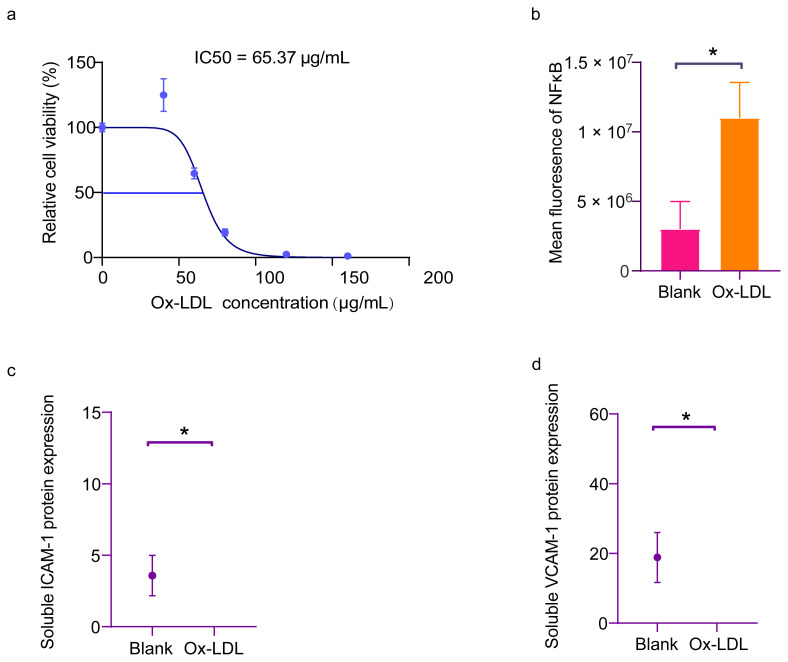
Ox-LDL-induced endothelial cell activation. (a) Dose-dependent effect of ox-LDL on cell viability in the RAECs. Cell viability was measured after the RAECs were treated with 0–160 μg/mL of ox-LDL for 24 h via the CCK 8 assay. The data in each group were normalized with the group treated with 0 μg/mL ox-LDL. (n = 3). The endothelial cell activity was 50% at a concentration of 65.73 μg/mL ox-LDL. (b) Comparison of the mean immunofluorescence intensity of the intracellular NF-κB nuclear translocations between the Blank group and ox-LDL group (n = 4). The ECs in the ox-LDL group were treated by 60 μg/mL of ox-LDL for 24 h. (c,d) Quantification of the ICAM-1 and ICAM-1 protein expression via ELISA between the Blank group and ox-LDL group (n = 6). * p < 0.05. Error bars represent the standard deviation of a separate sample.

**Figure 3:**
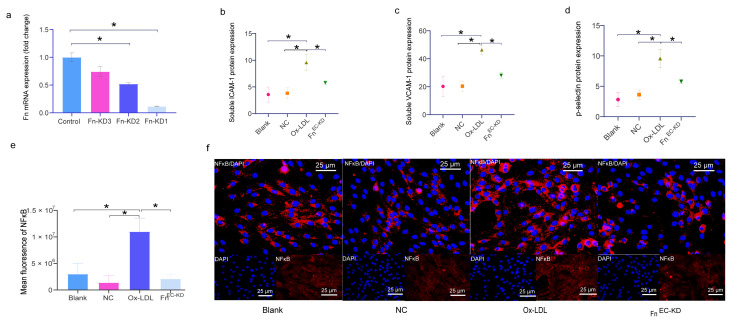
Fn deficiency in the endothelial cells inhibits endothelial cell activation induced by ox-LDL. (a) The expression levels of Fn in the RAECs transfected with 3 different Fn-targeted shRNAs (Fn-shRNA1, Fn-shRNA2, and Fn-shRNA3) or the control shRNA were analyzed via qPCR, corresponding to the Fn-KD1, Fn-KD2, Fn-KD3, and control group, respectively. (b–d) Quantification of the ICAM-1, VCAM-1, and p-selectin expression via ELISA (n = 6). (e,f) Immunofluorescence assay and quantification of the NFκB nuclear translocations (n ≥ 3). Representative images are shown at 400X magnification. Bars: 25 μm. The NC group was treated with the control shRNA. The ox-LDL group was treated with 60 μg/mL of ox-LDL for 24 h. The Fn^EC-KD^ group was transfected with the Fn-targeted shRNA and then treated with 60 μg/mL of ox-LDL for 24 h. * p < 0.05. Error bars represent the standard deviation of a separate sample.

Ox-LDL has been shown to promote endothelial cell activation via the TLR4/MyD88 pathway by activating the inflammatory transcription NFκB [5,6]. Before determining the concentration that would activate the endothelial cells, first, the concentration of ox-LDL was tested. At a concentration of 65.73 μg/mL of ox-LDL, endothelial cell activity was 50% (Figure 2a). Considering the proximity of the experimental concentration to 60 μg/mL, the endothelial cells were treated with ox-LDL at that specific concentration. Following the ox-LDL treatment, a notable increase in the nuclear translocation of NFκB p65 (Figure 2b) and secretion of endothelial cell activation markers, such as ICAM-1 and VCAM-1, was observed in the cell culture supernatants (Figures 2c and 2d). Based on the findings, it was concluded that ox-LDL induced endothelial cell activation. Consequently, 60 μg/mL of ox-LDL was selected as the intervention condition for endothelial cells. Experiments were conducted to optimize the knockdown efficiency of Fn-shRNA1 for Fn suppression in the AECs, utilizing qPCR analysis of the Fn mRNA in the endothelial cells (Figure 3a) as a basis for selection. The Fn mRNA levels in the Fn-shRNA1-transfected endothelial cells were 0.12 times higher than those in the control group. Next, ox-LDL was added to the Fn^EC-KD^ and the NFκB p65 activation, and ICAM-1, VCAM-1, and p-selectin expressions were examined to see if the Fn derived from the AECs had an effect on the ox-LDL-induced endothelial cell inflammation. The protein levels of the VCAM-1, ICAM-1, and p-selectin were then determined. It was seen that the ox-LDL significantly increased the VCAM-1, ICAM-1, and p-selectin expression in the endothelial cells, whereas Fn knockdown significantly reduced the ox-LDL-induced VCAM-1, ICAM-1, and p-selectin expressions in the endothelial cells (Figures 3b–3d). Significant disparity in the NFκB nuclear translocation was observed among the groups, as indicated by the study findings. While the ox-LDL intervention notably augmented the NFκB p65 nuclear translocation, the suppression of Fn in the AECs exhibited a significant inhibitory effect on the ox-LDL-induced NFκB p65 nuclear translocation (Figures 3e and 3f). In the AECs, the knockdown of Fn effectively suppressed the ox-LDL-induced NFκB activity and the expression of inflammatory factors, including VCAM-1, ICAM-1, and p-selectin.

### 3.2. Knockdown of AEC Fn inhibits vasoconstrictor secretion

**Figure 4:**
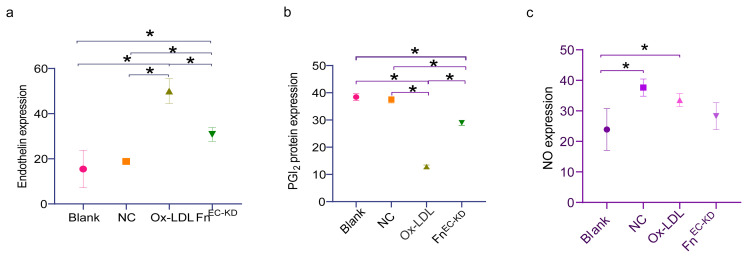
Fn deficiency in the endothelial cells inhibits the vasoconstrictor secretion induced by ox-LDL. (a,b) Quantification of the endothelin (n = 6), PGI2 (n = 3), and protein expression via ELISA between the groups. (c) Quantification of the NO expression via the biochemical assay (n = 3). The NC group was treated with the control shRNA. The ox-LDL group was treated with 60 μg/mL of ox-LDL for 24 h. The Fn^EC-KD^ group was transfected with the Fn-targeted shRNA and then treated by 60 μg/mL of ox-LDL for 24 h. * p < 0.05. Error bars represent the standard deviation of a separate sample.

Endothelial dysfunction is characterized by impaired vasodilatory capacity. Accordingly, ELISA was employed to quantify the endothelin and PGI2 levels in the cell culture supernatants. The ox-LDL stimulation increased endothelin, while decreasing PGI2 secretion in the AECs (Figures 4a and 4b). Nevertheless, the knockdown of Fn resulted in a significant reduction in endothelin secretion (Figure 4a), coupled with a significant increase in PGI2 expression (Figure 4b). These findings suggest that Fn derived from AECs improves vasodilatory dysfunction.

The level of NO in the culture supernatant was quantified, as NO serves as a well-known vasodilator with antiatherosclerotic effects, synthesized by NO synthase (NOS) [22]. When compared to the ox-LDL intervention alone, the negative vector and the ox-LDL intervention both resulted in a significant increase in NO secretion, whereas the Fn^EC-KD^ resulted in a slight decrease in NO production (Figure 4c).

### 3.3. pFn inhibits ox-LDL-induced endothelial cell activation

**Figure 5:**
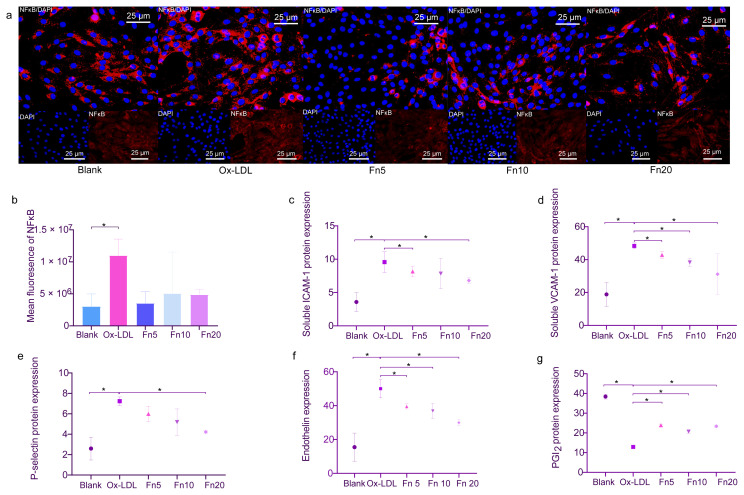
pFn inhibits endothelial cell activation induced by ox-LDL. (a,b) Immunofluorescence assay and quantification of the NFκB nuclear translocations (n ≥ 3). Representative images are shown at 400X magnification. Bars: 25 μm. (c–e) Quantification of the ICAM-1 (n = 6), VCAM-1 (n = 5), and p-selectin (n = 5) expression with ELISA between the groups. (f,g) Quantification of the endothelin (n = 6), and PGI2 (n = 3) protein expression via ELISA between the groups. The ox-LDL group was treated with 60 μg/mL of ox-LDL for 24 h. The Fn5, Fn10, and Fn20 groups were treated with 5, 10, and 20 μg/cm^2^ of pFn prior to the ox-LDL treatment. * p < 0.05. Error bars represent the standard deviation of a separate sample.

Hepatic cells are the primary source of pFn synthesis, it remains to be investigated whether pFn can also induce endothelial cell activation. Prior to the addition of ox-LDL, the AECs were pretreated with 3 distinct concentrations of pFn, at 5, 10, and 20 μg/cm^2^. Following treatment of the endothelial cells with various concentrations of pFn, the inhibition of the ox-LDL-induced NFκB p65 nuclear translocation was observed to be mild (Figures 5a and 5b).

Nevertheless, treatment with pFn at concentrations of 5 and 20 μg/cm^2^ significantly suppressed ox-LDL-induced ICAM-1 secretion in the intervention cells (Figure 5c). Furthermore, the intervention cells treated with pFn at concentrations of 5, 10, and 20 μg/cm^2^ exhibited significant inhibition of VCAM-1 secretion induced by ox-LDL, whereas the cells cultured with 20 μg/cm^2^ of pFn displayed the inhibition of ox-LDL-induced p-selectin secretion (Figures 5d and 5e). These findings suggest that pFn reduces the inflammation caused by ox-LDL in endothelial cells. Endothelin and PGI2 secretion were also investigated (Figures 5f and 5g). Distinct concentrations of pFn demonstrated significant inhibition of ox-LDL-induced endothelin secretion. The expression of PGI2 exhibited a notable increase at various pFn culture concentrations compared to the condition with ox-LDL intervention alone. These results indicate that pFn inhibits vasoconstriction.

## 4. Discussion

Endothelial cell activation is a cellular phenotype change characterized by increased permeability and the upregulation of inflammatory factor expression [4]. The endothelial phenotype has been demonstrated to undergo activation through matrix remodeling within the AS region [23]. The temporary matrix protein Fn aids in the activation of NFκB in endothelial cells by blood flow shear or ox-LDL [15,24] and upregulates the expression of endothelial cell activation markers VCAM-1 and ICAM-1 [7,25]. These findings indicate that Fn acts as a stimulant for endothelial cell activation. it was demonstrated herein that ox-LDL induces NFκB activation, leading to augmented secretion of VCAM-1, ICAM-1, and p-selectin in endothelial cells. Furthermore, the depletion of Fn from endothelial cells exerts inhibitory effects on ox-LDL-induced NFκB activation, as well as the secretion of VCAM-1, ICAM-1, and p-selectin. This observation aligns with the studies conducted by Orr et al. [25] and Funk et al. [19]. Moreover, the findings elucidated the indispensable role of Fn^EC^ in mediating the ox-LDL-induced activation of NFκB. Previous studies have suggested a link between NFκB activation in the AS region and the deposition of Fn [26]. In the present study, the reciprocal relationship between NFκB activation and Fn^EC^ deposition was determined. These findings suggest a mutually reinforcing mechanism where NFκB activation and AEC-derived Fn contribute to the activation of endothelial cells.

Nevertheless, Fn encompasses cFn originating from various cell types and pFn derived from hepatocytes, exhibiting distinct molecular structures. The Fn monomer consists of 3 different unitary repeats, FnI, FnII, and FnIII. FnIII exhibits alternative splicing, giving rise to Fn isoforms with or without the presence of extra region A (EDA) or extra region B (EDB) [27]. pFn, synthesized exclusively by hepatocytes, lacks the inclusion of EDA or EDB and is subsequently secreted into the bloodstream [28]; cFn is an extracellular matrix protein synthesized by cells such as endothelial cells, smooth muscle cells, and macrophages. It has been found that when cFn is knocked out, circulating pFn also fills the extracellular matrix of the arterial wall [21]. Hence, when a substantial deposition of Fn occurs within the AS region, it becomes crucial to determine which source of Fn specifically activates endothelial cells in this region. This aspect remains currently unresolved and requires further investigation.

Knockout studies targeting hematopoietic cell-derived Fn and liver-derived pFn in animal models have demonstrated a noteworthy reduction in plaque formation within rat vessels. Conversely, rats with solely hematopoietic cell-derived Fn knocked out displayed substantial lipid-containing plaque deposition [11]. These findings strongly suggest the involvement of liver-derived pFn in atherosclerotic plaque development. However, one study reported that the knockout of either Fn^EC^ or smooth muscle cell-derived cFn resulted in a decrease in the inflammatory cell population within AS plaques [12]. Moreover, it was observed that cFn, and not pFn, exacerbated cellular inflammation [7]. These findings suggest that cFn exhibits proinflammatory properties, while pFn appears to lack such effects. Herein, intervention with pFn resulted in a notable suppression of the ox-LDL-induced expressions of VCAM-1, ICAM-1, and p-selectin. The inclusion of pFn did not enhance the activation of NFκB induced by ox-LDL, implying that pFn does not contribute to the promotion of inflammation and endothelial cell activation by ox-LDL. Employing Fn-targeting shRNAs to disrupt the production of Fn transcripts from endothelial cells leads to a reduction in all Fn^EC^ isoforms. However, the experimental findings herein have shown that pFn inhibits endothelial cell inflammation and activation, whereas Fn derived from endothelial cells promotes these processes. These findings suggest that cFn derived from endothelial cells promotes ox-LDL-induced cellular inflammation and endothelial cell activation.

Platelet activation and decreased vasodilation caused by vasoconstrictors and inflammatory factors will cause severe constriction of the vascular lumen and increase the risk of thrombosis during the development of AS. When these pathologies occur in the coronary arteries, they cause severe myocardial supply shortages, eventually leading to myocardial necrosis. Activated endothelial cells secrete the powerful vasoconstrictors endothelin in the early stages of AS [29–31]. In order to maintain vascular dynamic homeostasis, the organism responds to vasoconstriction by releasing vasodilating cytokines such as PGI2 and NO [32]. Herein it was shown that the AECs activated by ox-LDL secreted large amounts of endothelin, which is in agreement with the above findings. Nonetheless, ox-LDL-induced endothelial cell activation significantly inhibited PGI2 secretion, implying that ox-LDL had severely impaired the compensatory capacity of the endothelial cells. Furthermore, knocking down the Fn in endothelial cells or adding pFn inhibited ox-LDL-induced endothelin bursts, while rescuing ox-LDL-inhibited PGI2 secretion. These observations suggest that pFn attenuates the activation of endothelial cells and mitigates endothelial dysfunction, playing a crucial role in preserving vasodilatory function. In contrast, endothelial cell-derived cFn is likely to contribute as a detrimental factor, exacerbating endothelial cell inflammation and inducing dysfunction. In future experiments, a knockout vector can be designed for cFn to transfect endothelial cells to further confirm the relationship between cFn and endothelial cell activation and endothelial dysfunction.

PGI2 and NO are both known vasodilators. Fn has been shown in studies to inhibit cellular NO production induced by altered shear stress [33]. In the present study, it was demonstrated that intervention with ox-LDL or a negative vector led to a significant increase in NO production. However, contradicting previous findings, knocking down the Fn did not result in significant up or downregulation of the NO production. Studies have demonstrated that minimal concentrations of ox-LDL can stimulate endothelial NOS (eNOS) to enhance NO synthesis [34]. This effect may be attributed to the inflammatory milieu, as the body strives to maintain homeostasis between vasoconstriction and diastolic relaxation. In the presence of elevated levels of the NFκB inducer, ox-LDL, it also triggers the activation of inducible NOS (iNOS) in cells, leading to the rapid production of substantial amounts of NO surpassing the rate of eNOS synthesis [35]. High levels of NO, accompanied by superoxide anion responses, are detrimental to cells [36] and may hasten endothelial cell activation and inflammation. Although the experimental data herein have not yet provided evidence for a correlation between endothelium-derived Fn and NO production, it was found that ox-LDL induced high cellular production of the NO as a consequence of unfavorable endothelial cell conditions.

In conclusion, the experiments conducted herein confirmed, at the cellular level, that pFn inhibits the expression of inflammatory factors and vasoconstrictor factors and facilitates the alleviation of the AS process. In contrast, endothelial cell-derived cFn has the potential to promote endothelial cell activation and endothelial dysfunction. These findings indicate that different Fn isoforms play distinct roles in the AS process, highlighting the contrasting effects of pFn and Fn^EC^ on AS promotion or inhibition. In order to elucidate the functional disparities between endothelial cell-derived cFn and pFn, our forthcoming experiments will involve the development of knockout mice specifically targeting pFn or cFn derived from endothelial cells. These investigations will provide valuable insights into the underlying mechanisms by which both forms of Fn contribute to AS.

## Figures and Tables

**Table 1:** Sequences of the shRNAs.

Name	Sequence
Fn-shRNA1 forward	5′TCGAGGCAGGATTGTCTATTCACCTTCAGTATTCAAGAGAGATACTGAAGGTGAATAGACAATCCTGTTTTTTA-3′
Fn-shRNA1 reverse	5′AGCTTAAAAAACAGGATTGTCTATTCACCTTCAGTATCTCTTGAATACTGAAGGTGAATAGACAATCCTGCC-3′
Fn-shRNA2 forward	5′TCGAGGCACCATCATGTGGACACACCTCCTAATTTCAAGAGAATTAGGAGGTGTCCACATGATGGTGTTTTTTA-3′
Fn-shRNA2 reverse	5′AGCTTAAAAAACACCATCATGTGGACACCTCCTAATTCTCTTGAAATTAGGAGGTGTCCACATGATGGTGCC-3′
Fn-shRNA3 forward	5′TCGAGGCGTGCCAGGATTACTGGCTACATTATTCAAGAGATAATGTAGCCAGTAATCCTGGCACGTTT- 3′
Fn-shRNA3 reverse	5′AGCTTAAAAAACGTGCCAGGATTACTGGCTACATTATCTCTTGAATAATGTAGCCAGTAATCCTGGCACGCC-3′
Control-shRNA1 forward	5′GATCCGTTCTCCGAACGTGTCACGTAATTCAAGAGATTACGTGACACACGTTCGGAGAATTTTTTC-3′
Control-shRNA1 reverse	5′AATTGAAAAAATTCTCCGAACGTGTCACGTAATCTCTTGAATTACGTGACACGTTCGGAGAACG-3′

**Table 2:** Primer sequences.

Primer	Sequences
Fn1 forward	5′-CCTTACACGGTTTCCCATTA-3′
Fn1 reverse	5′-TTTCCATTCCCGACAT-3′
Beta-actin forward	5′-GGAGATTACTGCCCTGGCTCCTA-3′
Beta-actin reverse	5′-GACTCATCGTACTCCTGCTTGCTG-3′

## Data Availability

The data used to support the findings of this study are available from the corresponding author upon request.
